# Effects of low-intensity pulsed ultrasound on the microorganisms of expressed prostatic secretion in patients with IIIB prostatitis

**DOI:** 10.1038/s41598-024-66329-x

**Published:** 2024-07-04

**Authors:** Wei-Jie Song, Ji-Wei Huang, Yuan Liu, Jie Wang, Wei Ding, Bin-Long Chen, Dong-Yi Peng, Zhi Long, Le-Ye He

**Affiliations:** 1 Department of Urology, The Third Xiangya Hospital, Central South University, No. 138 Tongzipo Road, Changsha City, 410013 Hunan Province China; 2 Sexual Health Research Center, The Third Xiangya Hospital, Central South University, Changsha, Hunan China

**Keywords:** Low-intensity pulsed ultrasound, IIIB prostatitis, High throughput, Second-generation sequencing, Expressed prostatic secretion, Microorganism, Bacteria, Clinical microbiology, Prostate

## Abstract

To detect and analyze the changes of microorganisms in expressed prostatic secretion (EPS) of patients with IIIB prostatitis before and after low-intensity pulsed ultrasound (LIPUS) treatment, and to explore the mechanism of LIPUS in the treatment of chronic prostatitis (CP). 25 patients (study power was estimated using a Dirichlet-multinomial approach and reached 96.5% at α = 0.05 using a sample size of 25) with IIIB prostatitis who were effective in LIPUS treatment were divided into two groups before and after LIPUS treatment. High throughput second-generation sequencing technique was used to detect and analyze the relative abundance of bacterial 16 s ribosomal variable regions in EPS before and after treatment. The data were analyzed by bioinformatics software and database, and differences with P < 0.05 were considered statistically significant. Beta diversity analysis showed that there was a significant difference between groups (P = 0.046). LEfSe detected four kinds of characteristic microorganisms in the EPS of patients with IIIB prostatitis before and after LIPUS treatment. After multiple comparisons among groups by DESeq2 method, six different microorganisms were found. LIPUS may improve patients’ clinical symptoms by changing the flora structure of EPS, stabilizing and affecting resident bacteria or opportunistic pathogens.

## Introduction

Chronic prostatitis (CP) usually refers to a prostate disease caused by various complex causes, with urethral irritation and chronic pelvic pain as the primary clinical manifestations. It is among the most common chronic diseases in young and middle-aged men^1^. Because it is difficult to treat and easy to relapse, it can severely affect the quality of life of patients and their mental status^2,3^. Unfortunately, the incidence of CP has been increasing in recent years. It has been reported that the incidence of CP in China has reached 6.0–32.9%^4^. Unfortunately, the pathogenesis of CP is still not very clear. Some scholars have recently found that CP may be closely related to some potential pathogenic microorganisms^5–7^. Currently, the clinical treatment of CP is still dominated by therapeutic medicines. Medication treatment can alleviate the clinical symptoms of patients to some extent. However, for some patients, especially IIIB prostatitis, the effect of medication treatment is poor, and the recurrence rate is high^8^. Consequently, the side effects of medication treatment are difficult to avoid and may sometimes even outweigh the potential therapeutic effect on the disease^9^. Therefore, as a new treatment for CP, clinicians, and patients are now considering low-intensity pulsed ultrasound (LIPUS). In recent years, many reports have been published on the efficacy and safety of LIPUS in treating IIIB prostatitis^10–12^. With the increasingly extensive application of LIPUS, people are beginning to pay more attention to its effective mechanism, which is of great significance not only for further promoting the application of LIPUS but also for understanding its possible side effects and its efficacy. Because of the added research on the relationship between pathogenic microorganisms and CP in recent years, we aimed to explore the possible mechanism of LIPUS in the treatment of IIIB prostatitis from the point of view of the effect of LIPUS on microorganisms in patients’ expressed prostatic secretion (EPS).

## Materials and methods

### Study population

Prior to study initiation, study power was estimated using a Dirichlet-multinomial approach and reached 96.5% at α = 0.05 using a sample size of 25^13^. In strict accordance with the classification criteria of prostatitis established by the National Institutes of Health (NIH), we selected 25 patients with IIIB prostatitis who were only effectively treated with LIPUS (the total score of NIH-chronic prostatitis symptom index (CPSI) decreased by four or more points) in Xiangya Third Hospital of Central South University from January 2022 to June 2022. All subjects were between the ages of 20 and 40 (CP/CPPS tends to occur more frequently in middle-aged and young adult males). This study was approved by the Ethics Committee of Xiangya Third Hospital of Central South University (Grant No. 22069), and the subjects' informed consent was obtained.

### Selection criteria

The inclusion criteria were as follows: (i) age was between 20 and 40 years old; (ii) NIH was classified as CPPS (IIIB), the clinical symptoms lasted more than 3 months, and the total score of CPSI was more than 10 points^9^; (iii) only received LIPUS treatment and regularly followed two courses, and the total CPSI score decreased to or more than four points; (iv) did not take any antibiotics within 3 months before LIPUS treatment; (v) the patient signed informed consent.

The exclusion criteria were designated as follows: (i) urinary tract infection; (ii) history of urinary cancer, surgery, radiotherapy, systemic chemotherapy; (iii) unilateral testicular pain, active urethral stricture or bladder stone with pelvic symptoms, or any other urinary disease associated with lower urinary tract symptoms, any neurological disease or disorder affecting the bladder; (iv) did not receive regular LIPUS treatment for two courses, or while receiving LIPUS treatment, took other therapeutic medicines or took antibiotics within 3 months before LIPUS treatment; (v) did not sign informed consent.

### Grouping and specimen collection

We analyzed symptoms of our patients before and after LIPUS treatment. During the treatment of LIPUS, the CP treatment mode was selected, and the patients were instructed to take the bladder lithotomy position and fully expose the perineum and pubic symphysis. The treatment head A and B of the therapeutic instrument were fixed in the perineum and pubic symphysis, respectively. The energy intensity was set at 1.25 w/cm^2^. Each treatment lasted for 10 min, once every other day, and five consecutive treatments were taken as a course of treatment. EPS samples of 25 subjects were collected before and after two courses of treatment in 30 min. Before collecting, we asked the patient to empty their bladder. We then cleaned the urethral orifice with sterile normal saline and wiped the residual liquid near the urethral orifice with aseptic gauze. Aseptically collected EPS samples were obtained by the same urologist from all patients included in the study. All specimens were stored in the refrigerator at − 80 ℃ immediately after collection.

### Instruments and reagents

The main instrument LIPUS therapeutic instrument model is LY-ED01, purchased from Hunan Lanyue Medical Technology Co Ltd. (Changsha, China); Nucleic acid electrophoresis instrument model DYCP-32C, agarose level electrophoresis instrument, purchased from Liuyi instrument Factory (Beijing, China); Covaris ultrasonic crusher Covaris S2 System, purchased from Massachusetts (USA); Qubit Fluorometric Quantification Qubit 2.0, purchased from Life Technologies (CA, USA); Bioanalyzer Agilent 2100, purchased from Agilent Technologies Co Ltd. (USA); The PCR instrument is T100PCR, purchased from Bio-Rad (USA); The sequencer is NovaSeq 6000, purchased from Illumina, San Diego (CA, USA), and the product purification kit is Qiagen gel recovery kit purchased from Qiagen Company (DUS, GER).

### Library construction

Total genomic DNA was extracted from EPS samples using the CTAB method. PCR amplified the V3+V4 variable region of 16 s ribosomal RNA (rRNA) gene with the primers 341F (5ʹ-CCTAYGGGRBGCASCAG-3ʹ) and 806R (5ʹ-GGACTACNNGGGTATCTAAT-3ʹ), respectively.

The samples were mixed with the same concentration according to the concentration of PCR products. Then the PCR products were purified by agarose gel electrophoresis with 1 × TAE concentration of 2%, and the target bands were recovered by tapping. We used the Illumina TruSeq^®^ DNA PCR-Free sample preparation kit library building kit (Illumina, USA) to build the library and add the index code. The constructed library was evaluated by Qubit quantification and library detection. Finally, the NovaSeq 6000 PE250 was used for sequencing, and the peer reads of 250 bp were obtained.

### Bioinformatics analysis

The analysis process was mainly done with reference to the “Atacama soil microbiome tutorial” tutorial in the Qiime2 documentation (https://docs.qiime2.org/2019.1/). The original sequence fastq file was imported by qiime tools import plug-in and converted into a file format that can be processed later by QIIME2. The QIIME2 dada2 plug-in was used to complete the quality control steps, pruning, denoising, splicing, and removing chimera, and finally, we obtained the feature sequence table^14^. Then the QIIME2 feature-classifier plug-in was used to compare the representative sequences of ASV to the pre-trained GREENGENES database with 99% similarity of version 13_8 (pruning the database to the area of V3V4 according to 341F/806R primer pairs), to get the species classification information table^15^. The QIIME2 feature-table plug-in removed all contaminated mitochondrial and chloroplast sequences. By using the “mixOmics” software package of R (v3.1.1) and partial least squares discriminant analysis (PLS-DA) multivariate statistical analysis method, according to the values of several variables observed or measured, how to classify the research objects and reveal the relationship between microbial communities and sample categories^16^. The diversity matrix is calculated by using the QIIME2 core-diversity plug-in. The Alpha diversity index evaluates the diversity degree of the sample itself at the feature sequence level. Notably, the Alpha diversity index includes observed operational taxonomic units (OUTs), Chao, Faith’s phylogenetic diversity, Shannon, and Simpson. The Beta diversity index evaluated the difference in microbial community structure among samples. Beta diversity index includes Bray Curtis and unweighted UniFrac and weighted UniFrac index, and the results are shown by PCoA and NMDS maps^17^. After obtaining the overall Beta diversity index, we combined the grouping information. The PERMANOVA and ANOSIM methods were used to compare whether there were significant differences in microbial composition and structure among different sample groups. Microorganisms with characteristics and different abundance among groups were identified by LEfSe and DEseq2 methods^18,19^. LEfSe method is based on the relative abundance table, a combination of nonparametric test and linear discriminant analysis and is suitable for the flora abundance difference test. It can find the characteristic microbes of each group (LDA > 2 & LDA > 4), that is, the microbes with high abundance in this group compared to other groups. The DESeq2 method can be used to find microbes with significant differences between groups by making multiple comparisons between groups. Unless otherwise noted, the parameters used in the above analysis are default settings.

### Ethics approval and consent to participate

The study was conducted in accordance with the Declaration of Helsinki, and approved by Institutional Review Board (IRB) of The Third Xiangya Hospital of Central South University (Grant No. 22069). Along with the subjects’ informed consent was obtained.

## Results

### Patients’ information

A total of 25 patients with IIIB prostatitis treated with LIPUS were enrolled in this study. The total score of CPSI was more than 10 points before treatment and decreased by four or more points after treatment (Table 1).

**Table 1:** Characteristics of patients in each group.

Cohort characteristics	After treatment	Before treatment
Age (year)	29.00 (27.00–34.50)	29.00 (27.00–34.50)
Body mass index (kg/m^2^)	23.37 (20.62–24.80)	23.37 (20.62–24.80)
Pain score median (IQR)	3.00 (1.00–6.50)	6.00 (4.00–8.50)
Urination score median (IQR)	3.00 (1.50–4.00)	7.00 (5.50–8.00)
Quality score median (IQR)	6.00 (4.00–7.00)	9.00 (7.50–11.00)
Total score1 median (IQR)	5.00 (4.00–8.50)	11.00 (10.00–15.00)
Total score2 median (IQR)	11.00 (8.00–16.50)	21.00 (19.00–26.00)

Total score1 = pain score + urination score; Total score2 = total score1 + quality score.

IQR *interquartile range.*

### OTU abundance analysis

**Figure 1:**
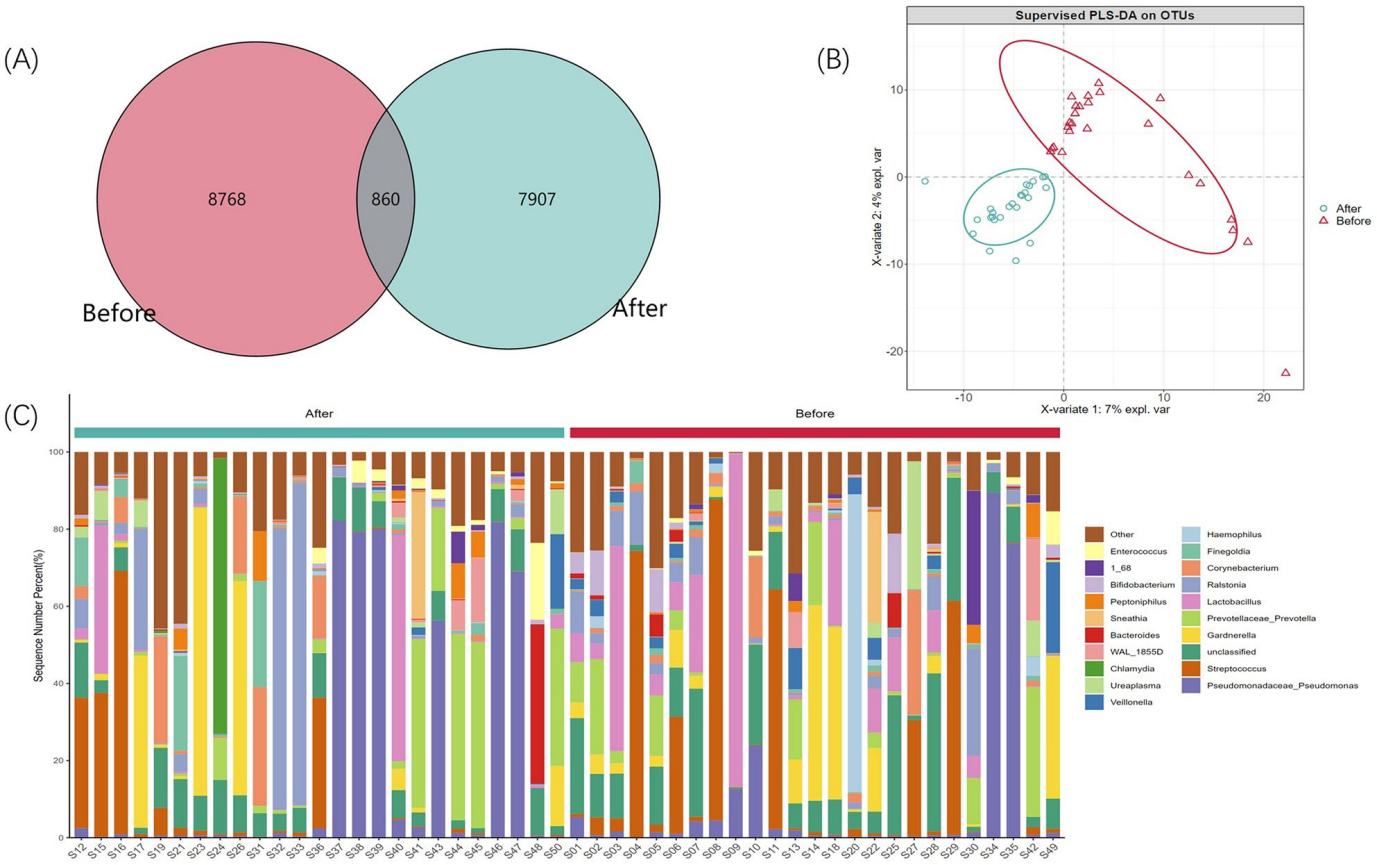
(**A**) Wayne diagram based on OUT. Show the common or unique number of OTU between different groups. Each ellipse represents a group, Before: before LIPUS treatment group; After: after LIPUS treatment group; (**B**) PLS-DA coordinate map. Each point represents a sample, the points of the same color belong to the same group, and the points of the same group are marked with an ellipse; (**C**) the relative distribution of each group at the genus level (the species in the top 20 of relative abundance). The illustration shows the 20 most dominant species at the genus level, and the remaining species with relatively low abundance are classified as other shown in the figure.

A total of 7,385,355 readable fragments were obtained from the valid data of 50 samples. After merging tag clustering, all valid sequences are clustered/denoised, and 17,535 OTUs were obtained according to 16 s rRNA data. We can find the unique or common OTUs by comparing the OTUs between samples. By drawing a Venn diagram to analyze the unique or common OTU between different sample groups, which directly shows the composition similarity and overlap of samples between groups at the OTU level (Fig. 1A). At the same time, we carried out a PLS-DA analysis, using the R language mixOmics package, of all OTU with an abundance greater than 10 and provided coordinate diagrams (Fig. 1B). From the Wayne diagram and PLS-DA analysis coordinate chart, it can be seen that there are differences in the structure of microflora in the EPS of patients with IIIB prostatitis before and after LIPUS treatment.

### Analysis of taxonomic annotations

The species annotation information was obtained by selecting the representative sequence of OTU and comparing it with the GREENGENES database (GREENGENES Database 13_8 version)^20^. Based on the species annotation information, the annotations were removed as chloroplasts, mitochondria, and OTU that cannot be annotated to the boundary level and the sequences they contain. Based on OUT’s absolute abundance and annotated information, 39 phylum, 100 classes, 162 orders, 236 families, 408 genera, and 245 species were annotated in this study. Figure 1C shows the distribution of each group’s top 20 species of relative abundance at the genus level. In contrast, Figs. S1, S2, S3, S4, S5 shows the relative distribution of the species in the top 20 of relative abundance in each group at the phylum, class, order, family, and species level, respectively.

### Heatmap analysis

**Figure 2:**
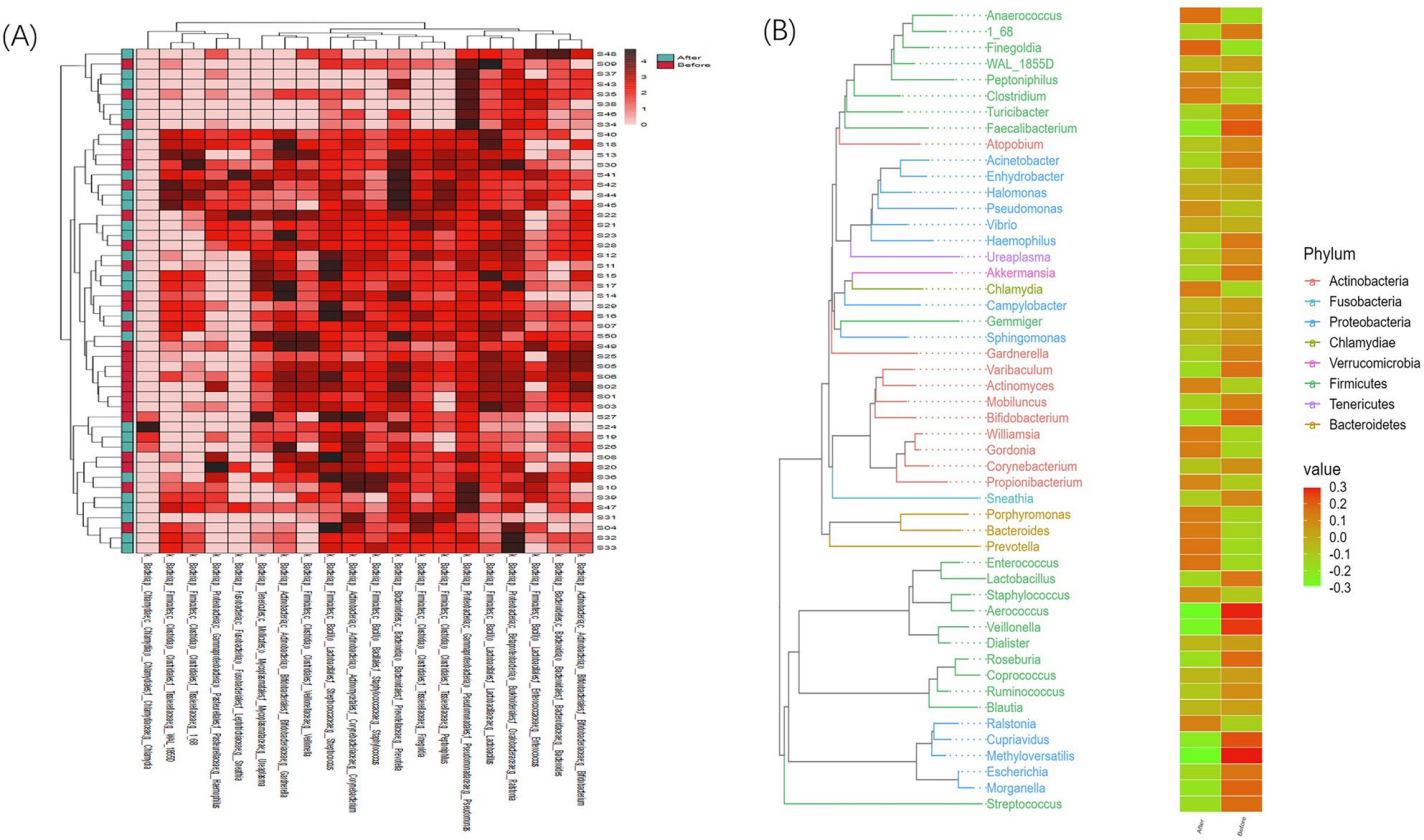
(**A**) A heat map of the genus level. Longitudinally is the sample name information. Horizontal is the annotation name of the genus horizontal classification. The clustering tree at the top of the figure is the similarity clustering of species abundance distribution in all samples. The clustering tree on the left is the similarity clustering of species abundance distribution of samples. The middle heat map is log10 (absolute abundance) heat map; (**B**) phylogenetic tree and inter-group abundance distribution heat map. On the left is the evolution tree. The branches of different colors represent different gates, each branch at the end represents an OTU, and the end notes the genus classification to which the corresponding OTU belongs. If there is no corresponding genus classification, it is expressed by unclassied genus, the heat map on the right is the standardized abundance, and the higher the value, the higher the relative abundance. Abundance standardization: the absolute abundance of each sample minus the average of the absolute abundance of the species is divided by the standard deviation so that the average abundance after standardization is 0 and the standard deviation is 1.

To study the similarity between different samples, the species concerned about the classification level (the first 20 of the default absolute abundance of species) are selected to realize sample clustering to investigate the similarities or differences between different samples or groups. We then carried on the horizontal clustering from the two aspects of classification information and the difference between samples to find the aggregation law of species or samples. In this study, heatmap cluster analysis was carried out at the phylum, class, order, family, genus (Fig. 2A), and species level. According to the 16 s rRNA sequence, the top five bacteria were *Pseudomonas*, *Prevotella*, *Streptococcus*, *Ralstonia*, and *Lactobacillus*. The phylogenetic analysis of the representative sequence of OTU was carried out by using the R language ggtree package (each genus selects an OTU with the highest abundance as the representative OTU, and then the top 50 genera with the highest abundance), the evolutionary tree was drawn, and the heat map was visualized with the absolute abundance of OTU in each group (Fig. 2B).

### Sample-based rarefaction analysis

**Figure 3:**
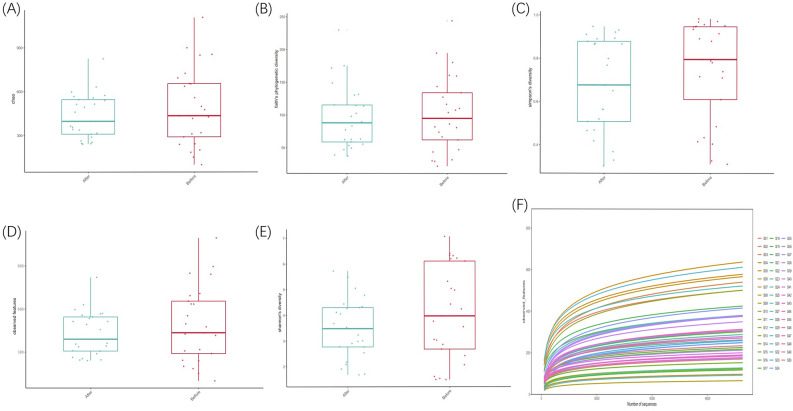
(**A**) box diagram of Chao index; (**B**) box diagram of Faith's phylogenetic diversity; (**C**) box diagram of Simpson index; (**D**) box diagram of observed features; (**E**) box diagram of Shannon index; (**F**) dilution curve of Alpha diversity index. The X axis extracts the sequence sequencing quantity in the graph, and the Y axis represents the corresponding Alpha diversity index. Each color represents a sample.

**Figure 4:**
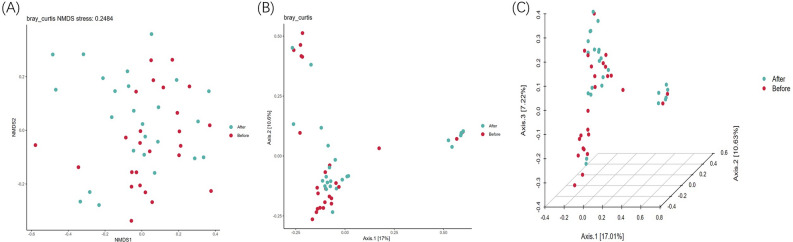
(**A**) NMDS analysis diagram based on Bray Curtis distance. PCoA is based on the distance matrix to find the principal coordinates, and the combination of principal coordinates with the greatest contribution is shown. The closer the distance of the sample is, the more similar the species composition and structure of the sample is; (**B**) 2D map of PCoA analysis based on Bray Curtis distance; (**C**) 3D map of PCoA analysis based on Bray Curtis distance.

Alpha diversity analysis was used to evaluate the microbial diversity of the sample itself, which was completed by the qiime2 diversity plug-in. The index of a single sample of sparse curves with different α diversity shows that the sequencing depth covers all species in the sample (Fig. 3F). Microbial diversity was described by observed features, Chao, Faith’s phylogenetic diversity, Shannon, and Simpson. After obtaining the overall Alpha diversity index, combined with grouping information, Wilcox test was used to compare the significant differences between groups accurately. The results showed no significant difference in species diversity between groups, as shown in Table 2. Figure 3A–E is a box chart of Alpha diversity between groups. Beta diversity analysis was used to evaluate samples’ differences in microbial community structure. Bray Curtis obtained the whole Beta diversity index, and the microbial community structure between groups was compared by the PERMANOVA method combined with grouping information. The results demonstrated a significant difference (P = 0.046), as shown in Table 3. Furthermore, Fig. 4A–C shows the groups’ Beta diversity PCoA and NMDS diagrams.

**Table 2:** Comparison of α‐diversity results among the groups (method: Wilcoxon).

α‐diversity index	Group1	Group2	P‐value
Chao	After treatment	Before treatment	0.56
Faith’s phylogenetic diversity	After treatment	Before treatment	0.82
Simpson’s diversity	After treatment	Before treatment	0.13
Observed features	After treatment	Before treatment	0.57
Shannon’s diversity	After treatment	Before treatment	0.30

Bold values indicate the statistically significant P values (P ≤ 0.05).

**Table 3:** Comparison of β‐diversity results among the groups (pairwise permanova results).

Group		Sample size	Permutations	Pseudo-F	P-value	q-value
After	Before	50	999	1.578	0.046	**0.046**

Bold values indicate the statistically significant q values (q ≤ 0.05).

q value is the corrected P value, which is of more reference value.

### LEfSe analysis

**Figure 5:**
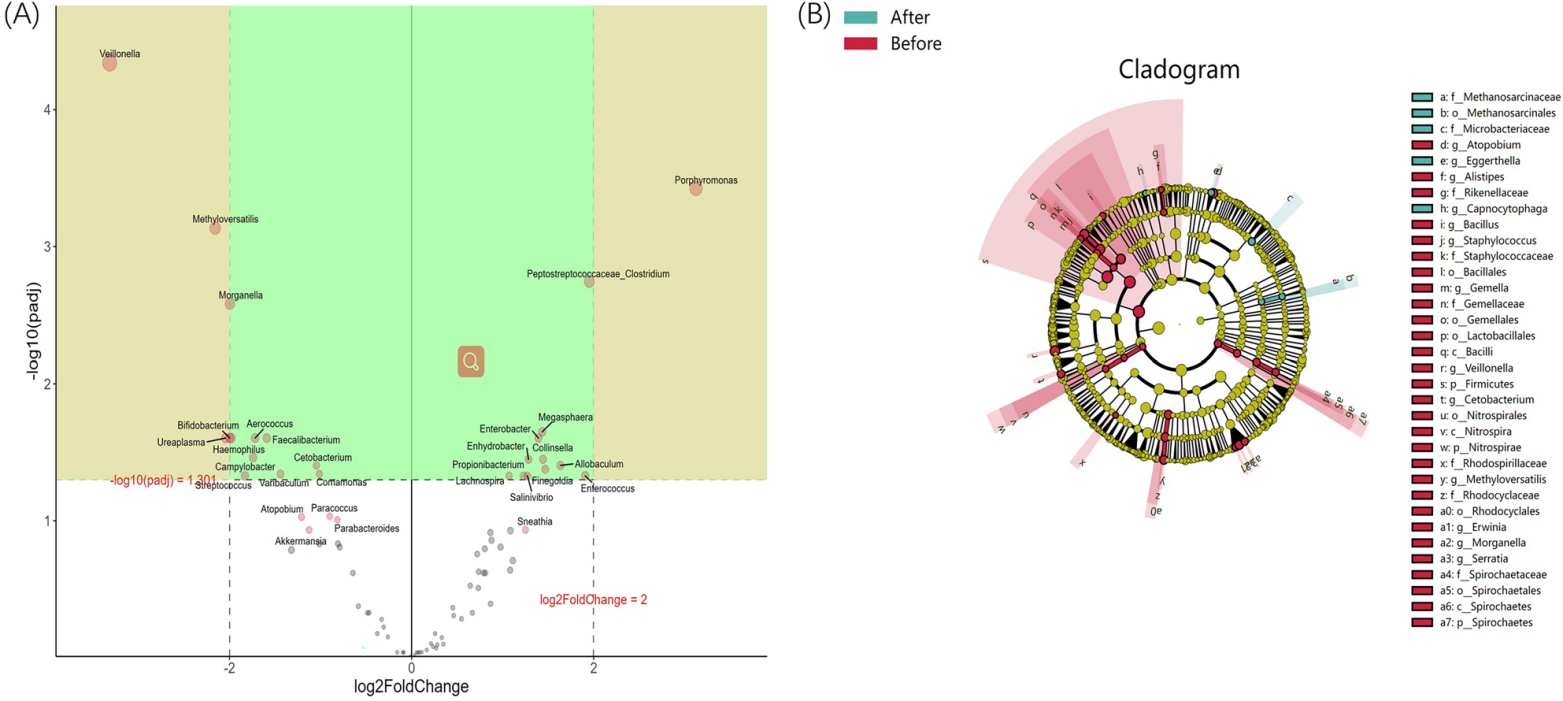
(**A**) DESeq2 analysis of volcano map; (**B**) LEfSe analysis cladogram diagram (LDA > 2). From inside to outside, cladogram diagrams correspond to different classification levels of families and genera, and the connections between levels represent the relationship of belonging. Each circle node represents a species. The yellow node signifies no significant difference between groups, and not yellow means that the species is the characteristic microorganism of the corresponding color group (the abundance is significantly higher in this group). The colored fan-shaped areas are marked with the sub-classification range of characteristic microorganisms.

LEfSe analysis showed that when LDA Score was equal to two at the genus level, 34 kinds of characteristic microorganisms were found in the EPS of patients before and after LIPUS treatment, including 29 before treatment and five after treatment (Fig. 5B). When the LDA score was equal to four at the genus level, four characteristic microorganisms were found in the EPS of patients with LIPUS before-treatment group, namely, *Lactobacillales*, *Bacilli*, *Veillonella*, and *Firmicutes* (Fig. S6). No characteristic microorganisms were found in the after-treatment group.

### Significance analysis of intergroup differences

By using the R language DESeq2 package, it was found that there were significant differences in six kinds of bacteria at genus level between patients before and after LIPUS treatment groups (log2FC > 2 & P < 0. 05). There were significant differences in *Veillonella*, *Methyloversatilis*, *Morganella*, *Bifidobacterium* and *Ureaplasma* in EPS before LIPUS treatment. There was a significant difference in *porphyromonas* in EPS after LIPUS treatment (Fig. 5A).

## Discussion

Prostatitis is the third most common disease in the genitourinary system. NIH classifies prostatitis into four types, of which type III chronic abacterial prostatitis/chronic pelvic pain syndrome (CP/CPPS) is the most common type, accounting for 80–90% of prostatitis^21^. According to EPS, semen, and urine samples after a prostate massage, CP/CPPS can be divided into inflammatory (IIIA) and non-inflammatory types (IIIB)^22^. Although no pathogens were found in CP/CPPS patients undergoing routine EPS bacterial culture, many researchers still consider infection as an important pathogenic factor. Mycoplasma, chlamydia, trichomonas, candida, viruses and parasites, and other pathogenic microorganism infections have been reported to be associated with the pathogenesis of CP/CPPS^23–25^.

At present, the primary treatment of CP/CPPS is medication therapy. Commonly used medicines in the clinic include α-receptor blockers, plant preparations, non-steroidal anti-inflammatory analgesics, and muscarinic receptor blockers. However, patients often report that the medication treatment is ineffective, the recurrence rate is high, and they are eager to receive more effective treatment^11^. It is under this background that LIPUS was born. The efficacy and safety of LIPUS in treating CP/CPPS, especially in the treatment of IIIB prostatitis, has been confirmed by many clinical researchers inland and abroad^1,11,12,26,27^. With the popularization and application of LIPUS, the effective mechanism of CP treatment has been paid more attention to by patients and doctors.

This study investigated 25 patients with IIIB prostatitis whose symptoms were significantly improved after LIPUS treatment. The microorganisms in their EPS before and after treatment were detected and analyzed. The results showed that *Pseudomonas, Prevotella*, *Streptococcus*, *Ralstonia,* and *Lactobacillus* were the most abundant bacteria in EPS. The structure and abundance of microflora in EPS of patients with IIIB prostatitis changed significantly before and after LIPUS treatment. The abundance of *Veillonella*, *Methyloversatilis*, *Morganella*, *Bifidobacterium*, *Ureaplasma*, *Lactobacillales*, *Bacilli*, and *Firmicutes* in EPS decreased significantly after LIPUS treatment (Fig. 5A and Fig. S6). It is important to emphasize that while our study results show the presence of multiple bacterial species in EPS from IIIB prostatitis patients, this is due to the use of advanced high-throughput sequencing technology. These bacteria would not have been detected through routine bacterial culture processes of EPS from IIIB prostatitis patients. As antimicrobial therapy was not clinically indicated, we solely utilized LIPUS as adjunctive therapy.

With the continuous development of detection technology, researchers have found microbial flora in many parts and organs of the human body. Notably, microbes have been detected in men’s urethra, prostate, and testes^28–30^. Microorganisms in human organs usually have unique flora structures and microecology^29^. As a colorless milky fluid continuously secreted by prostate epithelial cells, EPS can reflect the function and state of the prostate to some extent^31^. In support of this, Davidsson et al. found propionibacterium acne mainly in prostate cancer tissues and normal tissues^32^. Feng et al. found that prostate cancer tissues and normal tissues are rich in Escherichia coli, Propionibacterium acne, Acinetobacter, and Pseudomonas at the macro-genomic level^33^. Lee et al. isolated *Enterococcus faecalis* from EPS samples of CP patients^34^. Fang et al. used high-throughput sequencing technology to detect EPS samples from CP patients and normal healthy men and found a significant difference in the composition of the two flora as a whole^29^. Furthermore, we also detected a variety of bacteria in the EPS of patients, confirming the prevalence of microbial phenomena in EPS.

Interestingly, we found that the microflora in the EPS of the patients with IIIB prostatitis in the study changed significantly before and after LIPUS treatment groups and that the species and abundance of microorganisms decreased significantly after treatment. The reason for the analysis may be that some of the bacteria in EPS were destroyed by ultrasound produced by LIPUS. As early as 1927, Wood and others confirmed that ultrasound could have a fatal effect on microorganisms^35^. Follow-up studies also found that ultrasound can kill microorganisms, whether used alone or in combination with other means, whether high intensity or low intensity^36–38^. However, the patients with IIIB prostatitis in this study only received LIPUS treatment, and the symptoms of CP were significantly improved. Therefore, we hypothesize that during the treatment of IIIB prostatitis, some microorganisms in the EPS of patients with IIIB prostatitis are destroyed by the ultrasound emitted by LIPUS, resulting in the change of flora structure. Notably, this may be one of the reasons for the obvious improvement of CP symptoms after LIPUS treatment.

In this study, the EPS of patients was collected through urethral excretion. The microorganisms in EPS reflect the microorganisms in the prostate tissue and include some urethral colonization microorganisms. Importantly, we study EPS as a whole. Our research utilized advanced detection methods and scientific, statistical analyses methods to demonstrate that the structure and abundance of flora in EPS are changed following LIPUS treatment. However, for patients with CP/CPPS, it is not very clear whether there are specific pathogens in EPS and whether the changes in the structure and abundance of resident microflora or opportunistic pathogens in EPS are directly related to the clinical symptoms of patients. In theory, when the microecology of microflora in EPS changes under the action of some influencing factors, it may lead to the imbalance of normal flora or opportunistic pathogens, resulting in the occurrence and development of inflammation^7^. Following LIPUS treatment, some resident bacteria or opportunistic pathogens may be inhibited or eliminated to a certain extent, so the structure and abundance of bacteria in EPS tend to be balanced and stable. Thus, reducing the clinical symptoms of patients to a certain extent. However, the profound relationship between the structure and abundance of microflora in EPS and inflammatory factors and the pathogenesis of CP remains to be further studied. Moreover, normal microbial communities within individuals may vary due to differences in race, diet, culture, and other factors across regions. How the flora distribution of patients in different regions will change before and after LIPUS treatment needs to be further confirmed by multicenter collaborative research.

## Conclusion

In patients with IIIB prostatitis whose symptoms were significantly improved after LIPUS treatment, there were significant changes in the structure of flora in EPS before and after treatment. LIPUS may improve the clinical signs of patients with IIIB prostatitis by changing the flora structure of EPS, stabilizing and affecting resident bacteria or opportunistic pathogens.

### Supplementary Information

Supplementary Figures.

## Data Availability

The data presented in the study are deposited in the NCBI repository, accession number PRJNA1050891.
