# Supplementary material for: Effects of low-intensity pulsed ultrasound on the microorganisms of expressed prostatic secretion in patients with IIIB prostatitis
Source: Sci Rep. 2024 Jul 4;14:15368. doi: 10.1038/s41598-024-66329-x (PMC11224392; doi:10.1038/s41598-024-66329-x)
Supplement: Supplementary file 1 — Supplementary Figures. [file 41598_2024_66329_MOESM1_ESM.doc]

**
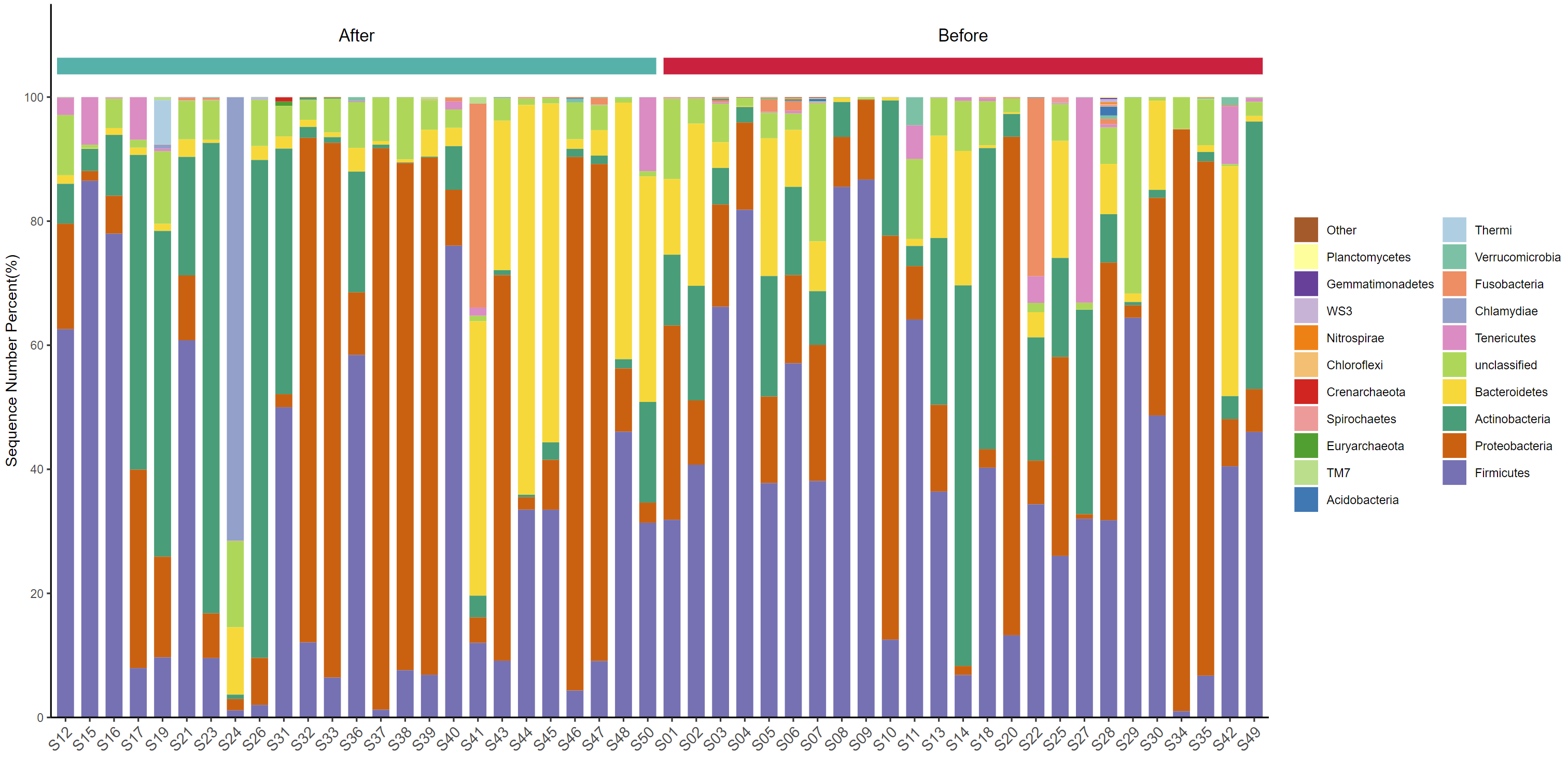
**

**FIGURE S1** The relative distribution of each group at the phylum level (the species in the top 20 of relative abundance). The illustration shows the 20 most dominant species at the genus level, and the remaining species with relatively low abundance are classified as Other shown in the figure.


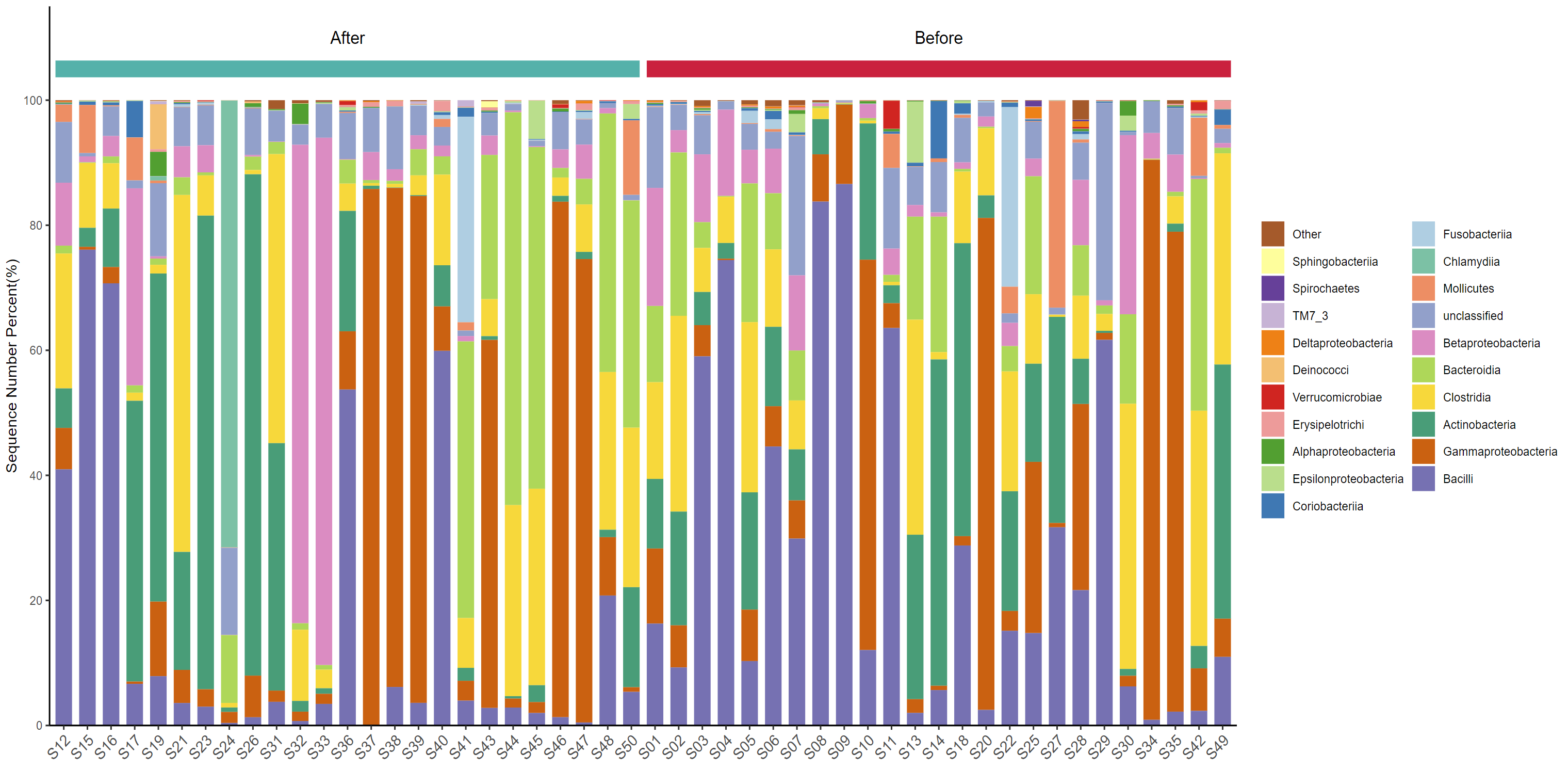


**FIGURE S2** The relative distribution of each group at the class level (the species in the top 20 of relative abundance). The illustration shows the 20 most dominant species at the genus level, and the remaining species with relatively low abundance are classified as Other shown in the figure.


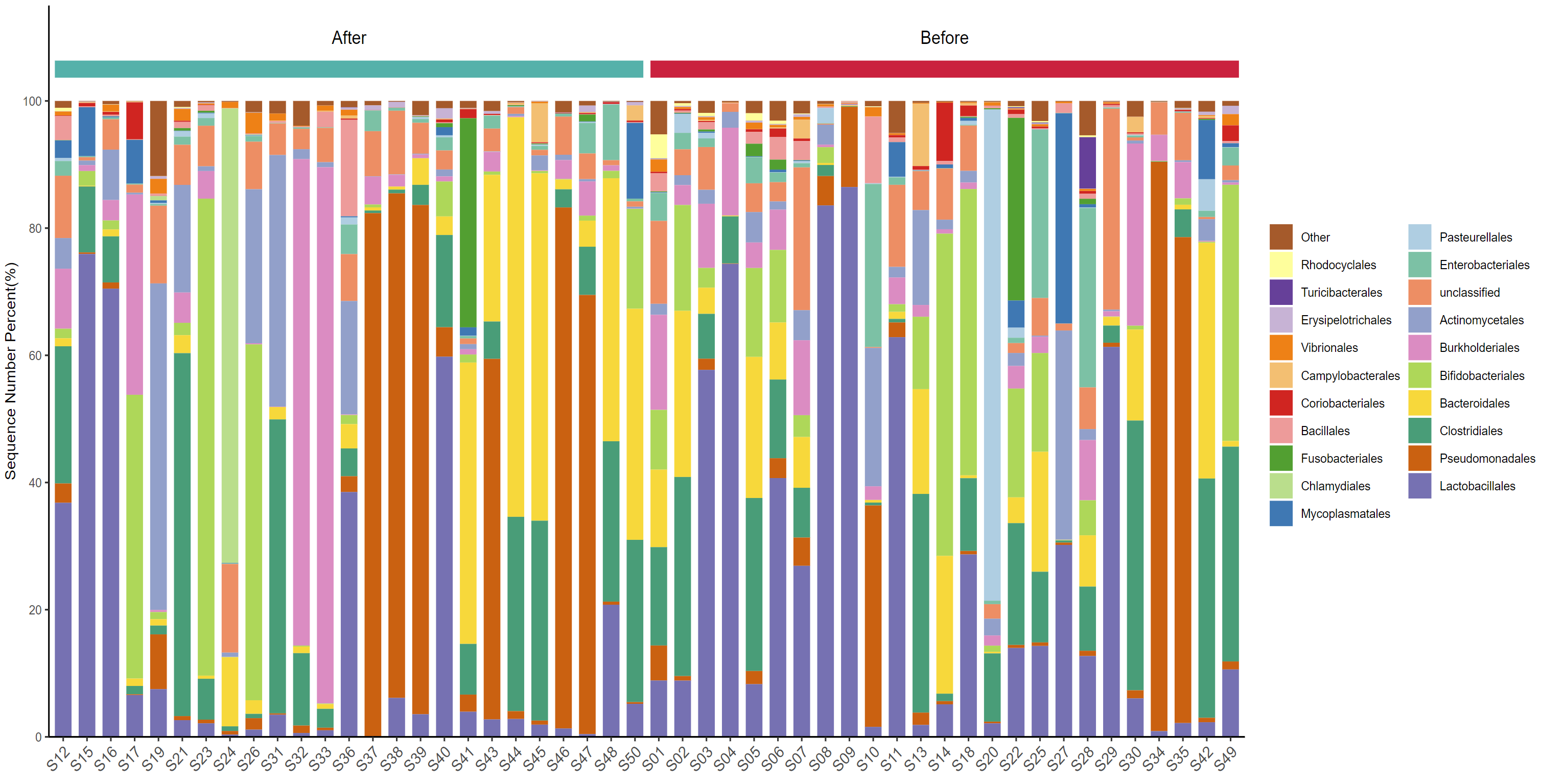


**FIGURE S3** The relative distribution of each group at the order level (the species in the top 20 of relative abundance). The illustration shows the 20 most dominant species at the genus level, and the remaining species with relatively low abundance are classified as Other shown in the figure.


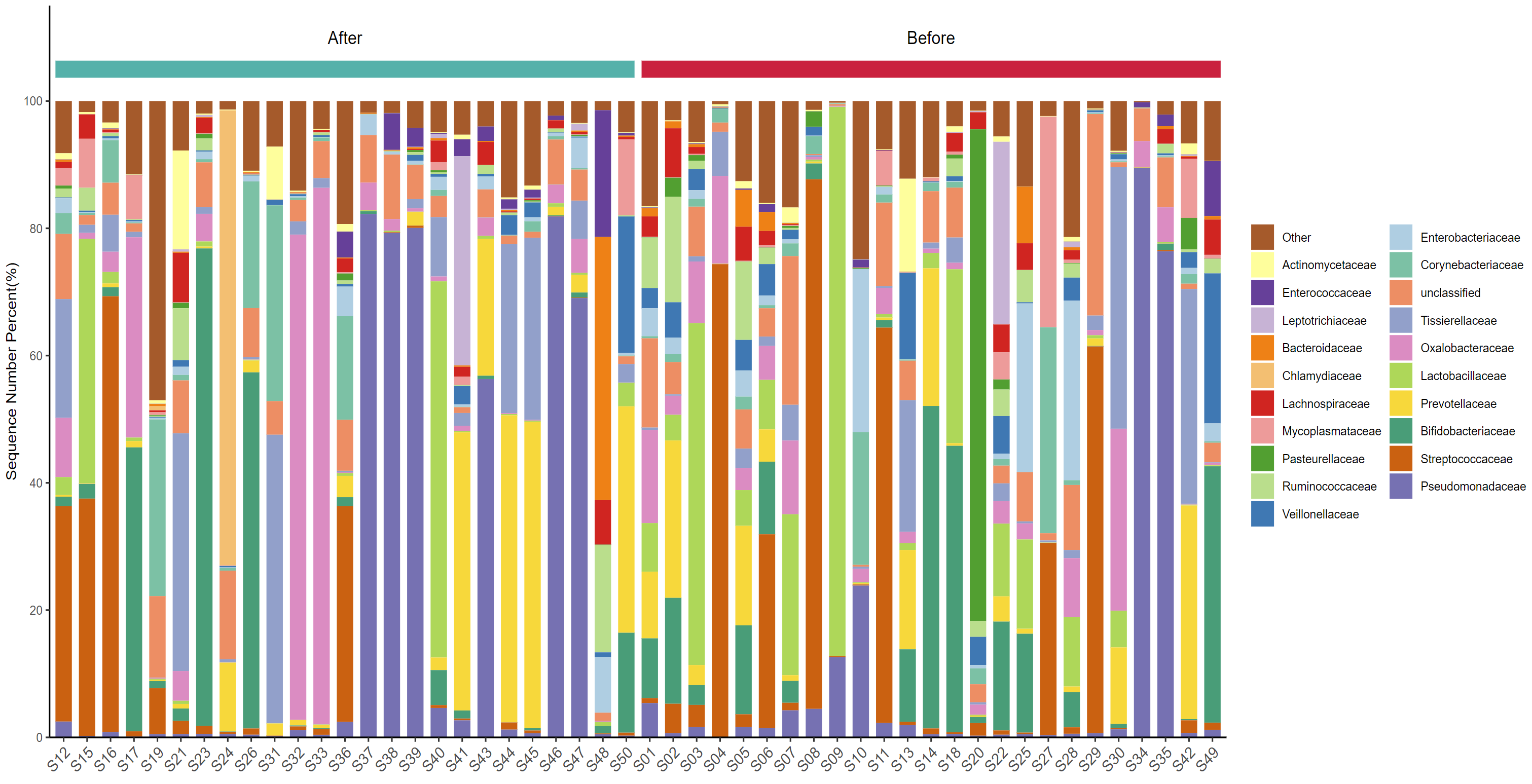


**FIGURE S4** The relative distribution of each group at the family level (the species in the top 20 of relative abundance). The illustration shows the 20 most dominant species at the genus level, and the remaining species with relatively low abundance are classified as Other shown in the figure.


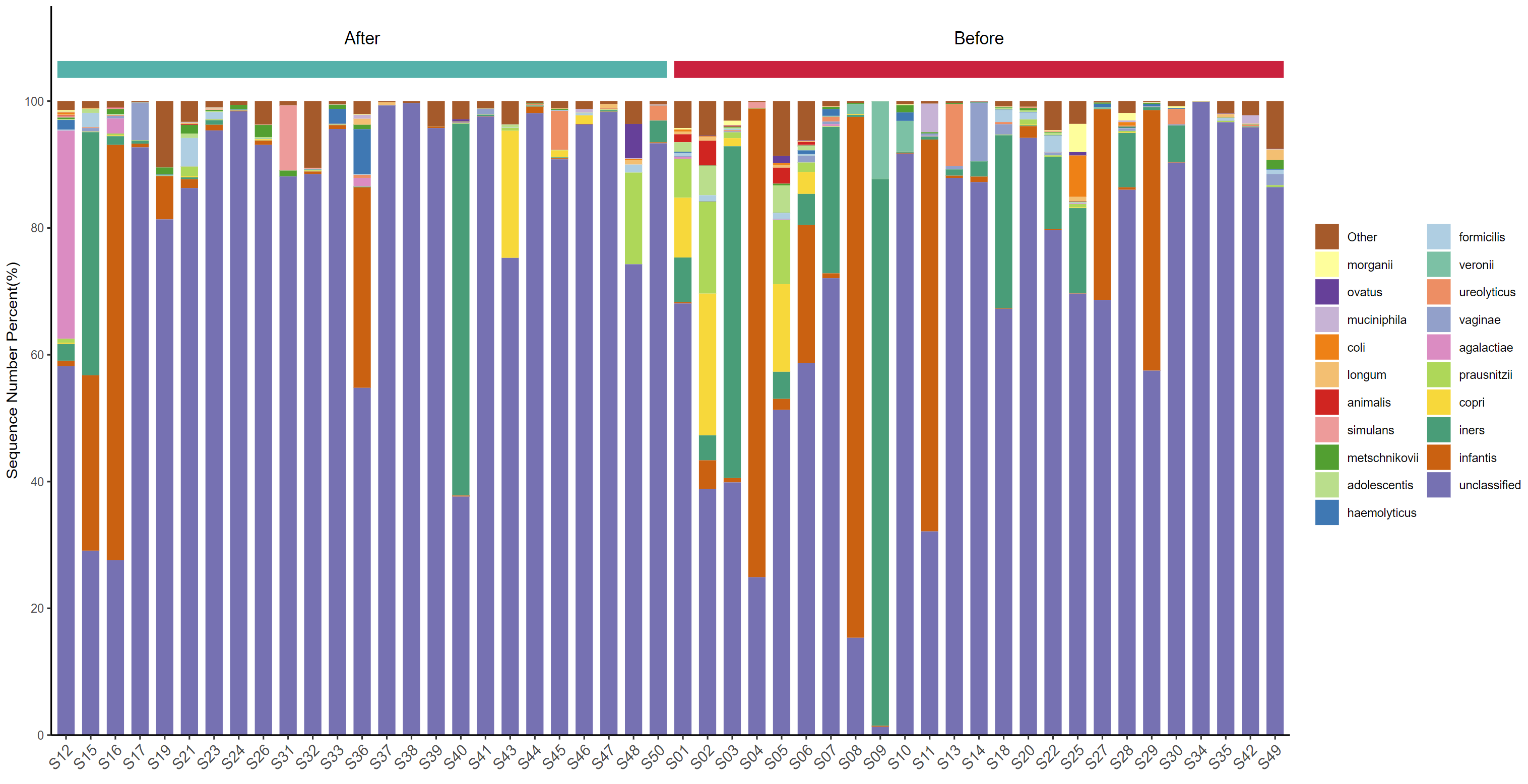


**FIGURE S5** The relative distribution of each group at the species level (the species in the top 20 of relative abundance). The illustration shows the 20 most dominant species at the genus level, and the remaining species with relatively low abundance are classified as Other shown in the figure.


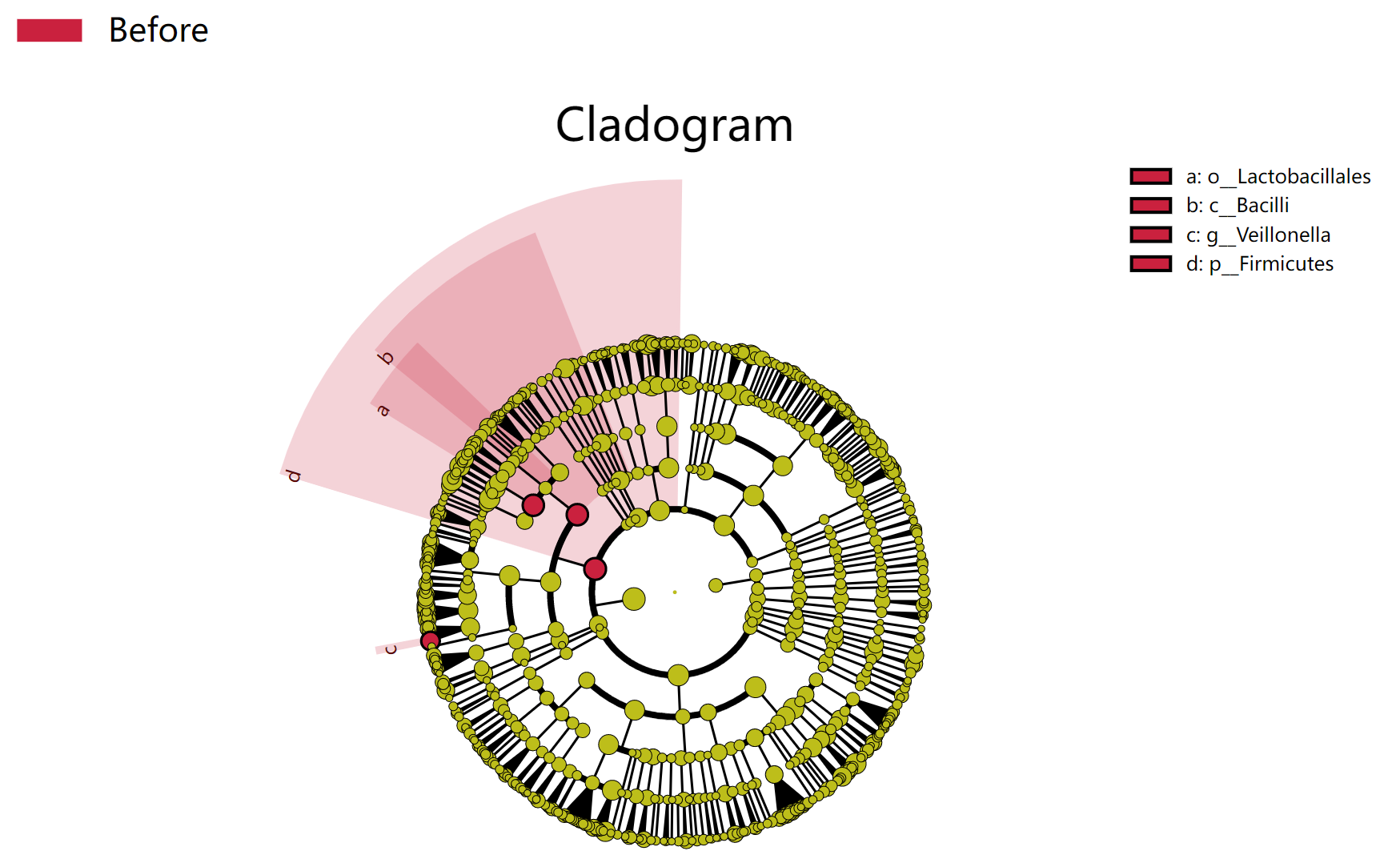


**FIGURE S6** LEfSe analysis cladogram diagram (LDA>4).
